# Impact of energy devices on the post-operative systemic immune response in women undergoing total laparoscopic hysterectomy for benign disease of the uterus

**DOI:** 10.4274/jtgga.2017.0076

**Published:** 2018-03-01

**Authors:** Kallol Kumar Roy, Netra GC, Seema Singhal, Juhi Bharti, Sunesh Kumar, Dipendra K. Mitra, Ruma Ray, Jyoti Meena, Perumal Vanamail

**Affiliations:** 1 Department of Obstetrics and Gynecology, All India Institute of Medical Sciences, New Delhi, India; 2 Department of Transplant Immunology and Immunogenetics, All India Institute of Medical Sciences, New Delhi, India; 3 Department of Pathology, All India Institute of Medical Sciences, New Delhi, India

**Keywords:** Total laparoscopic hysterectomy, immune response, cytokines, chemokines

## Abstract

**Objective:**

Laparoscopic surgery is associated with reduced surgical stress response, lesser post- operative immune function, and consequent early recovery compared with conventional open surgery. There is a lack of evidence regarding the inflammatory stress response with the use of different energy devices. The present study was conducted to evaluate and compare the inflammatory response in total laparoscopic hysterectomy (TLH) using three different energy devices.

**Material and Methods:**

A prospective randomized controlled study was conducted in 60 women with abnormal uterine bleeding undergoing TLH. They were divided into three groups based on the energy devices used, namely integrated bipolar and ultrasonic energy (Thunderbeat), ultrasonic (Harmonic) and electrothermal bipolar vessel sealing system (Ligasure). Cytokines and chemokines were measured in all three groups at different time points.

**Results:**

Serum levels of interleukin (IL)-6 and tumor necrosis factor-alpha (TNF-α) increased postsurgery in all three groups and gradually declined by 72 hours. The geometric mean serum (IL)-6 levels was highest with Ligasure at 24 hours as compared with the other groups. Levels of TNF-α, macrophage inflammatory protein (MIP-1) α, MIP-1 β were also higher at 3 hours in the Ligasure group. When the differences between the groups were measured at different time points, there was a significantly greater increase in serum IL-6 levels in the Ligasure group at 24 hours (p=0.010). No significant difference was found in the post-operative course between the groups.

**Conclusion:**

A greater inflammatory response was seen after the use of Ligasure indicating greater tissue damage. However, this response was not correlated with any difference in postoperative recovery.

## Introduction

Surgical trauma induces a stress response and leads to immunologic consequences. Surgical procedures stimulate a cascade of events that cause metabolic and inflammatory change. The extent and duration of the post-operative inflammatory response depends on the severity and type of intra-operative insult (1). Studies have reported that laparoscopic surgery was associated with reduced surgical stress response, lesser post-operative immune function, and consequent early recovery compared with conventional open surgery (2,3,4). Identification of markers of injury as a potential tool to predict perioperative outcomes remains an area of interest among researchers. Cytokines and chemokines are low-molecular-weight proteins produced by cells in the immune system in response to a variety of stimuli. They stimulate a cascade of events including the production and growth of lymphocytes, which in turn regulate the inflammatory response to surgical injury, affecting wound healing. Therefore, cytokines and chemokines serve as markers of operative stress response (5,6). It is imperative to understand the cytokine response to surgical trauma and the translation of this physiologic response into therapeutics is likely to optimize peri-operative care.

In endoscopic gynecologic surgery, the use of energy-based tissue sealing and cutting instruments has greatly advanced and facilitated complex laparoscopic procedures. Currently, various electrosurgical devices are commercially available such as advanced bipolar or ultrasonic devices (7,8). Due to the limited number of studies in the literature, there is insufficient evidence to recommend one energy source over another.

At present, there is lack of evidence regarding the inflammatory stress response with the use of different devices in cases of total laparoscopic hysterectomy (TLH). The present study was conducted to evaluate and compare the inflammatory response in terms of cytokines and chemokines in TLH using an integrated bipolar and ultrasonic energy device (Thunderbeat^TM^, Olympus, Japan), ultrasonic energy device (Harmonic, Ethicon Endosurgery, USA), and electrothermal bipolar vessel sealing system (Ligasure^TM^ V, Covidien, USA), which could explain a possible early recovery benefit of one energy source over another.

## Material and Methods

A prospective randomized controlled study (RCT) was conducted in the Department of Obstetrics and Gynecology over a period of one year (March 2015-April 2016), at the All India Institute of Medical Sciences, New Delhi. Ethical clearance from the Institutional Review Board was taken prior to commencement of the study and informed consent was obtained from all the participants. Sixty patients with abnormal uterine bleeding due to benign uterine diseases that was not responsive to medical therapy were recruited. Patients who had body mass index (BMI) of 20-30 kg/m^2^, uterine weight up to 300 grams, and uterine enlargement corresponding to 10 weeks gravid uterus were included in the study. Women with pelvic inflammatory disease, malignancy, more than one previous abdominal surgery, autoimmune diseases or history of anti-inflammatory drug use in the last one month were excluded. Sixty women who underwent TLH during study period were included and divided into three groups by adopting a randomization technique; Thunderbeat (group 1), Harmonic (group 2) and Ligasure (group 3) with 20 patients in each arm. To maintain an equal number of patients at any given time of the study, the block randomization technique was adopted. Accordingly, a total of 45 patients were recruited during a period of two months. These 45 patients were divided into 5 blocks, each containing 9 patients. In each block, randomization was performed in such a way that 3 patients were allocated randomly for each procedure. During the course of the study, 15 more patients were found to be eligible during a 3-month period. This block of 15 patients was randomized into three groups such that each group contained 5 patients. Block randomization was performed using Random Allocation Software version 1.0. All surgeries were performed by the same surgeon to avoid bias. A detailed examination of all patients was performed before the procedure, which included a hemogram, liver and renal function tests, blood sugar, chest X-ray, and electrocardiogram. Pre-operative endometrial aspirate was taken in all patients to rule out malignancy. Pre-operative ultrasound (transvaginal) was performed to estimate uterine weight based on length, width, and the anteroposterior diameter of the uterus. The inflammatory response in terms of both cytokines and chemokines induced by different energy devices was measured. The cytokines that were measured included interleukins (IL-6, IL-2, IL-17), tumor necrosis factor-alpha (TNF-α), interferon gamma (IFN-γ), and chemokines included chemokine ligand 5 [regulated on activation, normal T cell expressed and secreted (RANTES)] and macrophage inflammatory proteins (MIP-1 α, MIP-1 β). Venous blood samples were collected, serum was isolated, and stored at -70 °C. Serum cytokines and chemokines were then measured using a cytometric bead array assay. All patients underwent TLH under general anesthesia. Venous samples were collected pre-operatively (a) and post operatively at 3 hours (b), 24 hours (c) and 72 hours (d) in all patients to measure inflammatory mediators. A multiplexed cytometric bead array (BD, USA) was used to determine serum levels of pro-inflammatory cytokines and chemokines. This flow cytometric bead-based technology allowed simultaneous quantitation of multiple analytes. A constant one was added uniformly to all values before converting into log values to normalize the data log-transformation to avoid zero values because the data obtained were skewed. The geometric mean of log values was calculated as follows;

Geometric mean = Antilog (average)-1

In the post-operative period, all patients received slow intravenous analgesia in the form of tramadol 1.5 mg/kg body weight, eight-hourly for 24 hours, followed by oral tramadol as and when required. Patients were assessed for the return of gastro intestinal (GI) activity at 6 hours. Post-operative pain was assessed 24 hours after surgery using a visual analog scale (VAS) with scores of 0-10, where 0 represented no pain and 10 represented the worst pain possible. An assessment was also made for febrile episodes and other complications such as wound infection and vaginal bleeding.

### Statistical analysis

Data analysis was performed using the SPSS software (IBM, version 19.0). Descriptive measures such as mean, median, and standard deviation were computed for all continuous variables. A comparison of mean values between the groups was tested using one-way analysis of variance (ANOVA). Differences in serum parameters from pre-treatment to post-treatment time points were compared across the groups using the ANOVA test. Post-hoc pairwise comparison was performed using the Bonferroni test. In the event of non-normally distributed data, median values were compared using the non-parametric Mann-Whitney U test for two groups, and the Wilcoxon test for more than two groups. Frequency data by categories were compared using the chi-square/Fisher’s exact test as appropriate. For all statistical tests, p values <0.05 were considered as statistically significant.

## Results

**Figure 1:**
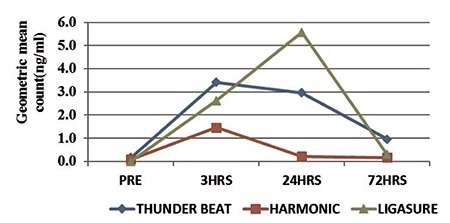
Geometric mean of serum interleukin-6 (ng/mL) among the three groups in the pre- and post-operative (3 hours, 24 hours, 72 hours) period

**Figure 2:**
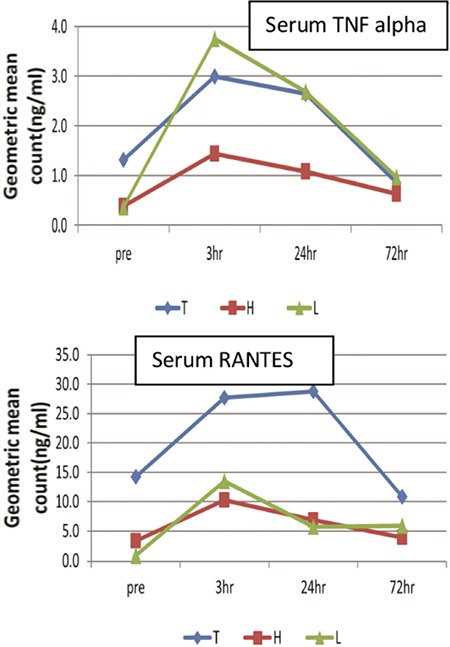
Geometric mean of serum tumor necrosis factor-α (ng/mL) and regulated on activation, normal T cell expressed and secreted (ng/mL) among the three groups in the pre- and post-operative (3 hours, 24 hours, 72 hours) period T: Thunderbeat; H: Harmonic; L: Ligasure; TNF: Tumor necrosis factor; RANTES: Regulated on activation, normal T cell expressed and secreted

The baseline characteristics of three groups were comparable with respect to age, BMI, uterine weight, indication for surgery, history of previous surgery, mean surgical duration, and length of hospital stay (Table 1). The geometric mean values of the different cytokines and chemokines were measured at various time points (preoperative, 3 hours, 24 hours, and 72 hours) and a comparison was made between the three groups. It was observed that serum levels of IL-6 peaked at 3 hours postsurgery in both Harmonic and Thunderbeat techniques and gradually declined by 72 hours, whereas the geometric mean of serum IL-6 levels was highest in the Ligasure group at 24 hours (Figure 1). TNF-α levels and RANTES peaked at 3 hours and gradually declined by 72 hours with all three energy devices (Figure 2). The highest geometric mean value of TNF-α was observed with Ligasure at three hours.

**Figure 3:**
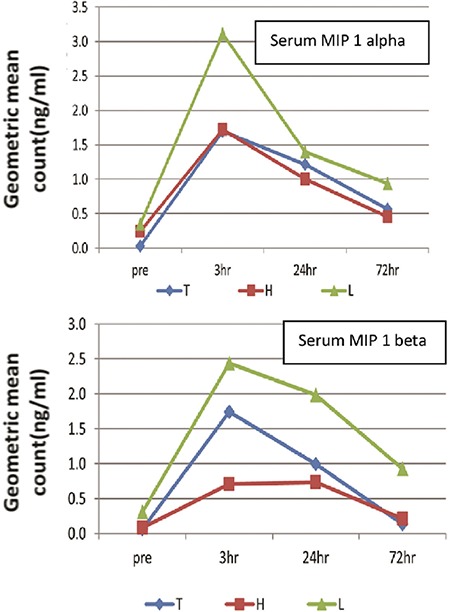
Geometric mean of serum macrophage inflammatory protein-1 α (ng/mL) and macrophage inflammatory protein-1 β (ng/mL) among the three groups in the pre- and post-operative (3 hours, 24 hours, 72 hours) period T: Thunderbeat; H: Harmonic; L: Ligasure; MIP: Macrophage inflammatory protein

Higher and sustained values of RANTES were observed with Thunderbeat as compared with the other two methods. The geometric mean values of MIP-1 α and MIP-1 β peaked at 3 hours and gradually declined by 72 hours. The highest geometric mean value of MIP-1 α and MIP-1 β was observed in the Ligasure method (Figure 3). This indicated greater tissue damage in the Ligasure group. 

To compare between the groups, differences in the geometric mean values were taken at various time points. There was a significantly higher increase in serum IL-6 levels in the Ligasure group as compared with Thunderbeat and Harmonic groups at 24 hours (p=0.010). This indicated that there was greater tissue damage in the Ligasure group. However, there was no significant difference between the three groups in terms of inflammatory response as seen in levels of TNF-α, RANTES, MIP-1 α, and MIP-1 β levels. This shows that there was equivalent tissue trauma in all three groups (Table 2). The levels of other markers (IL-2, IL-17 and IFN-γ) did not rise at the chosen time points postsurgery. Therefore, data could not be pooled for analysis.

The inflammatory response in the three groups was then analyzed in terms of clinical post-operative recovery. No significant difference was observed between the three groups in terms of return of GI activity at 6 hours [group 1: 17 (85%) vs. group 2: 18 (90%) vs. group 3: 16 (80%); p=0.676], fever [group 1: 0% vs. group 2: 1 (5%) vs. group 3: 3 (13%); p=0.153] and mean VAS score (group 1: 3.60±0.94 vs. group 2: 3.35±0.87 vs. group 3: 3.60±0.94; p=0.613). 

To summarize, each device lead to some amount of inflammatory response in terms of a rise in cytokines and chemokines in the immediate post-operative period; however, Ligasure produced a more sustained and greater inflammatory response at 24 hours postsurgery. However, this rise was not associated with any difference in the post-operative course.

## Discussion

Surgical trauma leads to an acute-phase response, mediated by cytokines, which are signaling peptides. Cytokines recruit reticuloendothelial cells (lymphocytes, monocytes and macrophages) and induce the production of chemokines to amplify the response. In turn, these mediators participate in the process of angiogenesis and wound repair (9,10). Cytokines balance the inflammatory and anti-inflammatory effects and an uncontrolled production of inflammatory cytokines can result in delayed recovery after surgery. Excessive cytokines also cause insulin resistance through a complex immunophysiologic response to surgery (5). Various cytokines such as IL-2, IL-6, IL-17, IFN-γ, and TNF-α, and chemokines such as RANTES, and MIP-1 α and MIP-1 β are released by activated macrophages during surgery. The hypothesis of enhancing postoperative recovery after surgery is partially based on limiting the release of cytokines from tissue injury (5,6).

Minimally invasive surgery is associated with reduced inflammatory response, and better preserved immune competence and recovery benefits in the post-operative period as compared with open surgery (2,3). Fretland et al. (4) compared the stress immune response in cases of laparoscopic and open resection of colorectal liver metastasis. The authors found significantly lower levels of inflammatory mediators [HMGB-1, cfDNA, IL-6, C-reactive protein (CRP), and MIP-1 β] (p<0.05) in the laparoscopy group compared with the open surgery group. Our previous randomized study compared surgical stress response after non-descent vaginal hysterectomy (NDVH) with laparoscopic-assisted vaginal hysterectomy (LAVH) in 20 women. The increment in levels of IL-6 from preoperative to 3-hour postoperative levels were analyzed and were significantly higher in the LAVH group (0.38 ng/mL) as compared with the NDVH group (0.06 ng/mL) (p=0.027). NDVH, which is associated with minimal surgical trauma and tissue handling, resulted in a lower inflammatory response (11).

The practice of endoscopy was revolutionized with the advent of modern multifunctional energy devices. Studies have evaluated the different available devices in terms of their safety, efficacy, extent of thermal injury, and versatility (7,8); however, none of the devices has been proven superior over another. In an attempt, Sietses et al. (12) compared an ultrasonic energy device with monopolar electrosurgery and analyzed the immune response evoked by each device. They measured changes in leukocyte count, CRP, and monocyte human leukocyte antigen – antigen D-related expression in cases of laparoscopic cholecystectomy. Both the ultrasonic energy device and monopolar diathermy resulted in a comparable stress immune response and therefore were found to be equally traumatic. However, it was speculated that surgical injury during lap cholecystectomy was insufficient to demonstrate a difference despite the expected advantage of the ultrasonic energy source over diathermy (12).

Our preliminary study shows novel observations regarding the differences in inflammatory response of IL-6, TNF-α, RANTES, MIP-1 α, and MIP-1 β for various energy sources and their impact on post-operative recovery in TLH. Serum levels of IL-6 at 24 hours were found to be significantly higher with Ligasure as compared with the Harmonic scalpel and Thunderbeat. This indicates that there is greater tissue trauma with Ligasure.

Of all the markers, IL-6 is the main acute-phase protein (5); its levels are an indicator of the extent of surgical trauma and thus can be considered as a predictor of morbidity after surgical intervention (4). IL-6 has been used as a parameter for the extent of inflamed and damaged tissues and higher levels have been found in cases of open surgery than in laparoscopy (6,10). Previous studies have also shown that IL-6 levels rise and peak between 3 and 24 hours after surgery and return to baseline in 3-4 days, similar to our findings (10,13,14).

Studies with other cytokines and chemokines are lacking. Although there was no significant difference for other cytokines and chemokines between the three groups, the various cytokines and chemokines rapidly peaked between 3 and 24 hours after the surgical trauma and this transient increase in soluble factors declined in blood by 72 hours.

All the patients received tramadol in the post-operative period for analgesia. It has a dual mode of action; besides being a weak opioid agonist, it inhibits uptake of serotonin and noradrenaline. It is clearly evident from previous studies that it does not influence IL levels in the post-operative period (15). Non-steroidal anti-inflammatory drugs were not used to remove the confounding factor.

We found no differences in post-operative outcomes with the use of any of the devices, despite the difference in levels of inflammatory markers. To the best of our knowledge, no prior randomized controlled trial has addressed this issue in cases of laparoscopic hysterectomy. Available studies on the comparison of post-operative outcomes with use of various energy sources in different surgeries (thyroidectomy, colectomy) have inconsistent results. The majority of studies show no difference in outcomes; however, small sample sizes and the heterogeneity of the studies are the main limitations (16).

To our knowledge, this is the first RCT to study the impact of these three energy devices on the immune system in humans. However, the small sample size remains the main constraint and any conclusion regarding the superiority of one energy source over another in terms of immune response cannot be drawn. Larger studies are required for more definitive results.  

## Figures and Tables

**Table 1:**
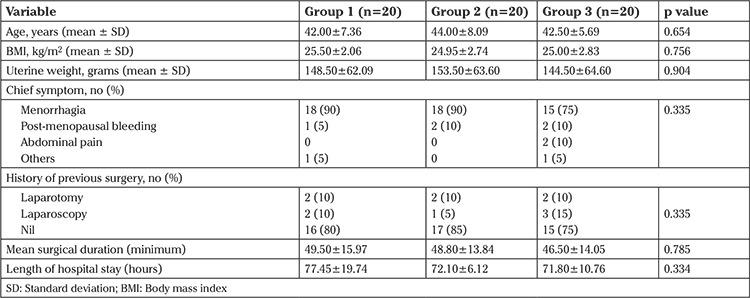
Clinical characteristics of the patients

**Table 2:**
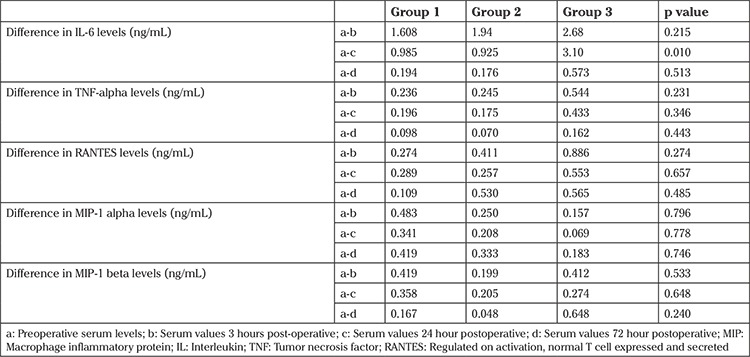
Comparison of cytokines and chemokines between patients in the three groups
